# Point-of-care human milk concentration by passive osmosis: comprehensive analysis of fresh human milk samples

**DOI:** 10.1038/s41372-024-01988-2

**Published:** 2024-05-17

**Authors:** Elizabeth R. Schinkel, Elizabeth R. Nelson, Jae H. Kim, Maryanne T. Perrin, Roger Dyer, Rajavel Elango, Lars Bode, David C. Dallas, Jiraporn Lueangsakulthai, Carrie-Ellen Briere, Sarah N. Taylor

**Affiliations:** 1 Mother’s Milk is Best Inc., R&D, 100 Business Park Drive, Unit #5, Tyngsboro, MA 01879 USA; 2 Perinatal Institute, Department of Pediatrics, Cincinnati Children’s Hospital Medical Center, University of Cincinnati College of Medicine, Cincinnati, OH 45229 USA; 3 Department of Nutrition, University of North Carolina Greensboro, Greensboro, NC 27412 USA; 4 Analytical Core for Metabolomics and Nutrition, British Columbia Children’s Hospital Research Institute, University of British Columbia, Vancouver, BC V5Z 4H4 Canada; 5 Department of Pediatrics, University of British Columbia, Vancouver, BC V6H, 3V4 Canada; 6 Bode Lab, Department of Pediatrics, School of Medicine, University of California, San Diego, San Diego, CA 92093 USA; 7 Dallas Lab, Nutrition Program, College of Health, Oregon State University, Corvallis, OR 97331 USA; 8 Briere Lab, Elaine Marieb College of Nursing, University of Massachusetts Amherst, Amherst, MA 01003 USA; 9 Institute for Nursing Research and Evidence-Based Practice, Connecticut Children’s, Hartford, CT 06106 US; 10 Division of Neonatology, Yale School of Medicine, New Haven, CT 06520 USA

**Keywords:** Paediatrics, Nutritional supplements

## Abstract

**Objective:**

Preterm infants need enrichment of human milk (HM) for optimal growth. This study evaluated a novel, point-of-care human milk concentration (HMC) process for water removal from fresh HM samples by passive osmotic concentration.

**Study design:**

Nineteen fresh HM samples were concentrated by incubation with the HMC devices for 3 h at 4 °C. Pre- and post-concentration HM samples were compared by HM properties for: pH, osmolality, macronutrients, enzyme activity, bioactive, and total cell viability.

**Results:**

Passive osmotic concentration reduced HM volume by an average of 16.3% ± 3.8% without a significant effect on pH or cell viability. Ten of the 41 HM components did not differ significantly (*p* > 0.05) between pre- and post-concentration samples. Twenty-three increased within the expected range by volume reduction. Six increased more than expected, two less than expected, and none decreased significantly.

**Conclusion:**

Passive osmotic concentration of fresh HM can concentrate HM components by selective removal of water. HM osmolality and pH remained within neonatal feeding parameters.

## Introduction

Mother’s own milk (MOM) has unique nutritional and health benefits for preterm infants and reduces neonatal morbidity, mortality, and NICU costs in a dose-dependent manner [1, 2]. Feeding preterm infants, MOM improves brain, vision, microbiome, and immune system development and reduces the incidence of bronchopulmonary dysplasia (BPD), retinopathy of prematurity (ROP), necrotizing enterocolitis (NEC), and neonatal sepsis [3–6]. MOM is a complex, biologically active form of nutrition; its composition fluctuates due to maternal hormonal and dietary influences. Artificially replicating MOM will be incredibly complicated, predictably expensive, and not foreseeable in the near future [1–8]. Consequently, increasing MOM intake by preterm infants is a public health priority [7–13].

Even when abundantly available, MOM is generally not the sole nutrient source for preterm infants due to their high nutrient needs and lower volume tolerances [14–16]. Most NICUs in the United States fortify MOM and donor human milk (DHM) with bovine milk-derived fortifiers, which are the only readily available, low-cost options to achieve adequate extrauterine growth [9, 10, 16]. However, the use of bovine milk-derived fortifiers in preterm infant feeding may impact human milk components and the risk of morbidities [4, 9, 10, 17]. Donor human milk (DHM)-derived fortifiers are also available but are more limited in use due to their higher cost, concerns about ethical sourcing, and a lack of proven efficacy [15]. Recently DHM-derived fortifiers were also linked to an increased incidence of hypoglycemia compared with feeding with bovine milk-derived fortifiers [18]. Moreover, DHM-derived fortifiers displace as much as 50% of MOM to achieve a caloric density adequate for preterm infant growth [19]. Thus, despite the challenge of providing adequate nutrients, feeding preterm infants fresh MOM should be prioritized for optimal growth [1–8, 11–14].

**Fig. 1:**
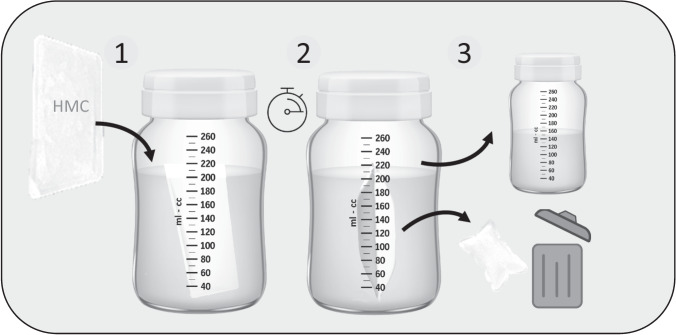
Human Milk Concentration Device steps of use. (1) Place HMC device in MOM, (2) HMC device passively absorbs water, (3) HMC device is removed, and concentrated milk is fed to a preterm infant.

Passive osmotic concentration is a novel point-of-care approach to increasing the nutrient and bioactive content of MOM that avoids heat and pressure damage and displacement of MOM. This process relies on osmotic draw across a limited permeable membrane to remove only the smallest molecules (<0.0007 micron), such as water, from HM. The specific embodiment of such an approach is a single-use human milk concentration (HMC) device composed of an osmotic membrane packet that is added to fresh or thawed HM that leads to passive concentration of HM components outside the device by removal of a defined amount of water (Fig. 1). To ensure broad accessibility and implementation, the HMC device was developed with attention to standard NICU feeding workflows, cost limitations and is compatible with standard 50- to 240-mL breast pump bottles. The HMC device may be used while HM is stored under refrigeration or being warmed for feeding. This HMC device has been shown to increase the concentrations of macronutrients and bioactive molecules in samples of previously frozen, pasteurized, and unpasteurized HM [20]. That proof-of-concept study was performed on previously frozen donor HM without available data on lactation stage, storage age, or reason for donor bank rejection for use. The HM sample sets were analyzed after a concentration at temperatures equivalent to milk storage or feeding preparation in US NICUs. (4 °C/refrigeration, 20 °C room temperature or 37 °C HM warming temperature) and results compared to baseline HM nutrient analysis.

The current study hypothesized that in fresh, never frozen/thawed HM samples; the sensitive HM components avoid damage from applied heat or pressure and increase when concentrated via passive osmosis using the HMC device. To test this hypothesis, the pH, osmolality, enzymatic activity, and concentrations of macronutrients, fatty acids, lactose, sodium, IgA, and lactoferrin in fresh HM samples were analyzed at baseline and after passive osmotic concentration with the HMC device. The impact of passive osmotic concentration on the viability of cells in fresh HM was also assessed.

## Materials/subjects and methods

### Human milk collection and concentration

The New England IRB approved the study protocol. Participants were recruited by distributing informational flyers, cooperating with Mother’s Milk Bank Northeast volunteer coordinators, and approaching Baby Café^TM^ participants who self-identified as having a surplus milk supply sufficient to donate a milk sample of 60–140 mL. Informed consent was obtained.

Fresh HM samples were collected within 30 min to 20 h (avg 11.5 h) after expression. Prior to collection, the samples were stored by the donors at 4 °C. The time after expression was self-reported by the donors. Before removing aliquots for baseline analysis and passive osmotic concentration, the HM samples were each gently swirled to mix the contents. The aliquot removed for baseline analysis of unconcentrated HM was stored at 4 °C.

Passive osmotic concentration was performed as follows. First, the HMC device was rinsed with filtered water warmed to 38 °C and placed in 75 mL of fresh HM (*V*_initial_) in an 80-mL (polypropylene, non-BPA) volume-marked bottle. Next, the bottle was capped and stored at 4 °C for 3 h to allow passive osmotic concentration of the HM. Finally, the HMC device was removed, and the masses of the HM (*V*_final_) and the HMC device were determined [20]. Analyses of the cell viability and energy, fat, carbohydrate, and protein contents of the concentrated milk were performed immediately. The remaining matched HM samples (unconcentrated and concentrated) in their respective containers were each gently swirled to combine their contents, then aliquoted and shipped overnight on dry ice to individual laboratories for other analyses.

The concentration (i.e., volume reduction) of milk was calculated using the following formula:

$${\frac{{\Delta }_{{vol}}}{{V}_{{initial}}}x100={HM}}_{{con}}$$

where $${\Delta }_{{vol}}={{{\mid{V}_{{final}}-{V}_{{initial}}{\mid}}}}$$

### Human milk macronutrients by mid-infrared analysis

The concentrations of energy, fat, carbohydrates, protein (crude), and true protein in the HM samples were analyzed using a Miris Human Milk Analyzer (Miris HMA, Uppsala, Sweden) according to the published device protocol [21]. The samples were stored at 4 °C before analysis. In brief, the samples were heated to 40 °C in a Miris heater (Miris, Uppsala, Sweden) and homogenized (Miris Ultrasonic Processor, Uppsala, Sweden). Each measurement required 2 mL of HM, and the samples were analyzed in duplicate. The Miris HMA reports crude protein so true protein was determined by assuming that 20% of the crude protein measurement is attributable to non-protein nitrogen [22].

### Human milk component analysis

The concentrations of protein, lactose, lactoferrin, active IgA, and sodium in HM were analyzed using unconcentrated and concentrated HM samples shipped overnight on dry ice. The samples were received frozen and were stored at –80 °C until analysis, which was performed immediately after thawing. Protein concentration was assessed in triplicate using the bicinchoninic acid (BCA) assay (Cat. No. PI23225, Fisher Scientific, Waltham, MA, USA) as previously described [23]. The average coefficient of variation (CV) for protein concentration was 4.0% (range, 0.7–9.3%). The lactose concentration was measured in triplicate using enzymatic methods (K-LACGAR, Megazyme, Bray, Ireland) after removal of protein and fat using Carrez I and II solutions (SC9101 and SC9102, Fisher Scientific) and filtering. This methodology is based on the methods of AOAC 2006.06 and has been validated in HM [24]. The average CV for lactose content was 2.2% (range, 0.2–9.3%). The lactoferrin concentration was determined in triplicate using a commercial enzyme-linked immunosorbent assay (ELISA) kit for human lactoferrin (Cat. No. EL2011-1, AssayPro, St. Charles, MO, USA) [25]. The average CV for lactoferrin concentration was 3.5% (range, 0.7–11.4%). IgA activity against *E. coli* antigens was measured in triplicate by ELISA, as previously described [26]. The average CV for active IgA concentration was 3.9% (range, 0.8–8.5%). The sodium concentration was measured in duplicate using a benchtop ion-selective electrode system (Orion Dual Star Dual Channel Benchtop Meter with Orion ROSS Sodium Combination Electrode, Thermo Fisher Scientific, Waltham, MA, USA) [27]. The average CV for sodium concentration was 0.1% (range, 0.0–0.6%).

### Human milk enzymatic activity analysis

Enzymatic activity in HM was analyzed using unconcentrated and concentrated HM samples shipped overnight on dry ice. The samples were thawed at 4 °C and centrifuged at 4250 × *g* for 10 min at 4 °C. The infranatant from below the upper-fat layer was collected by pipette and stored in 400 µL aliquots at −80 °C until use in assays. The aliquots were thawed only once to avoid possible enzyme degradation during thawing and freezing. Commercial assay kits were used to measure the activities of lysozyme (Cat. No. K236-100, BioVision, Milpitas, CA, USA), platelet-activating factor (PAF) acetylhydrolase (Cat. No. K765-100, BioVision), catalase (Cat. No. K773-100, BioVision) and glutathione peroxidase (Cat. No. K762-100, BioVision) according to the manufacturers’ instructions. The HM samples were used at 1× and 2× dilutions for lysozyme activity assays, 4× and 8× dilutions for PAF acetylhydrolase activity assays, 50× and 100× dilutions for catalase activity assays, and 400× and 800× dilutions for glutathione peroxidase activity assays.

Bile salt-stimulated lipase (BSSL) activity was assayed as described by Koh et al. [28] with some modifications. HM samples were used at 2× and 4× dilutions. A standard curve was constructed by serial dilution of *p*-nitrophenol to obtain a concentration range of 23.44 to 750 µM. The production of *p*-nitrophenol from *p-*nitrophenyl myristate was measured in a spectrophotometer by monitoring the absorbance at 405 nm every 30 s for 10 min at 37 °C.

### Human milk oligosaccharide analysis

Nineteen HM oligosaccharides (HMOs) were selected for analysis. These HMOs were selected because their absolute concentrations can be measured via separation and identification on an HPLC column with chemically defined standards used as a reference. These HMOs were analyzed using a previously published protocol [29]. The HMOs analyzed were: 2’-fucosyllactose (2’-FL), lacto-*N*-difucohexaose I (DFLNT), lacto-*N-*hexaose (LNH), disialyllacto-*N*-tetraose (DSLNT), 3-fucosyllactose (3FL), lacto-*N*-neotetraose (LNnT), sialyl-lacto-*N*-tetraose c (LSTc), disialyllacto-*N*-hexaose (DSLNH), 3’-sialyllactose (3’-SL), fucosyllacto-*N*-hexaose (FLNH), difucosyllacto-*N*-hexaose (DFLNH), difucosyllactose (DFLAC), lacto-*N*-tetraose (LNT), sialyl-lacto-*N*-tetraose b (LSTb), fucosyl-disialyllacto-*N*-hexose (FDSLNH), 6’-sialyllactose (6’SL), lacto-*N*-fucopentaose I (LNFP I), lacto-*N*-fucopentaose II (LNFP II), lacto-*N*-fucopentaose III (LNFP III).

### Small molecule analysis

The concentrations of free choline, phosphocholine, betaine, phosphatidylcholine (PC) and sphingomyelin (SPH) were determined by high-performance liquid chromatography tandem mass spectrometry (HPLC-MS/MS) using stable isotope-labeled internal standards. Free choline, phosphocholine and betaine were analyzed simultaneously as described previously [30]. The quantitative HPLC-MS/MS assay was highly reproducible for free choline and betaine measurement, with inter- and intra-assay CVs of ~3%. However, the quantitative HPLC-MS/MS assay was not highly reproducible for phosphocholine measurement, with inter- and intra-assay CVs of <12%. PC and SPH were analyzed separately using a modification of the method of Lindahl et al. [31]. The CVs were 4% for PC analysis and 10% for SPH analysis.

### Fatty acid analysis

Fatty acid content was analyzed by gas chromatography (GC) following the method of Lepage and Roy [32]. An Agilent 6850 gas chromatograph equipped with a flame ionization detector (FID), an SP2380 column (15 mm × 0.25 mm), and Agilent ChemStation software were used. Hydrogen was used as the carrier gas. To determine fatty acid content, the area counts of individual fatty acids were compared to the area counts of known quantities of nonanoic acid (C9:0) and heptadecanoic acid (C17:0), which were added to the HM samples as internal standards. The inclusion of internal standards also enabled the correction of the detector response for short-chain fatty acids of 12 carbons or fewer. The total fatty acid content was calculated by summing the contents of the individual fatty acids. For quality control of the total fatty acid and individual fatty acid content measurements, one milk sample was analyzed 5 times on the same day to determine the intra-assay variability, and the same sample was analyzed on 4 additional days to determine the inter-assay variability. The quantitative GC-FID assay for total fatty acids was highly reproducible, with inter- and intra-assay CVs of <2%.

To determine the linearity of the assay over a range of fatty acid concentrations, the contents of individual fatty acids and total fatty acids in different volumes of one milk sample were determined. The relationship between HM sample volume (i.e., total fatty acid quantity) and total fatty acid content was linear between ~3.75 and 6.1 g/dL (*r*^2^ = 0.996).

### pH and osmolality analysis

The pH and osmolality of HM were analyzed using concentrated and unconcentrated HM samples that had been shipped on dry ice and stored at −80 °C. After thawing, the pH of the sample was measured with a SevenCompact S220 pH/ion meter (Mettler-Toledo) equipped with a combined sealed glass electrode. The electrode was equilibrated before the pH value was recorded. Osmolality was measured in duplicate using an osmometer (Model 3320, Advanced Instruments, Norwood, MA, USA) after calibrating the machine with a 290 mOsm standard. If duplicate readings differed by more than 3 mOsm, a third reading was taken. Only one sample required a third reading.

### Human milk cell viability analysis

Cells were isolated from unconcentrated and concentrated HM samples using the protocol of Hassiotou et al. [33]. Briefly, the HM samples were diluted 1:2 with PBS and centrifuged at 800 × *g* for 20 min. The fat layer and supernatant were removed, and the cell pellet was resuspended in 1–2 mL of PBS and centrifuged again. After another round of resuspension in PBS and centrifugation to wash the cells, the supernatant was removed, and the cells were resuspended in a small volume of PBS. Finally, the cells were counted using trypan blue exclusion in an automated cell counter (Countess, Thermo Fisher). Samples were processed pairwise, and the percentages of live and dead cells were compared between unconcentrated and concentrated HM samples.

### Statistical analysis

All data were analyzed using JMP Version 17.2. Outliers were identified using a robust fit by Huber M-Estimation to estimate the center and spread (*K* Sigma = 4). Paired *t* tests were used to determine if differences in the concentrations of components were statistically significant (*p* < 0.05).

## Results

### Volume reduction of human milk by passive osmotic concentration

**Fig. 2:**
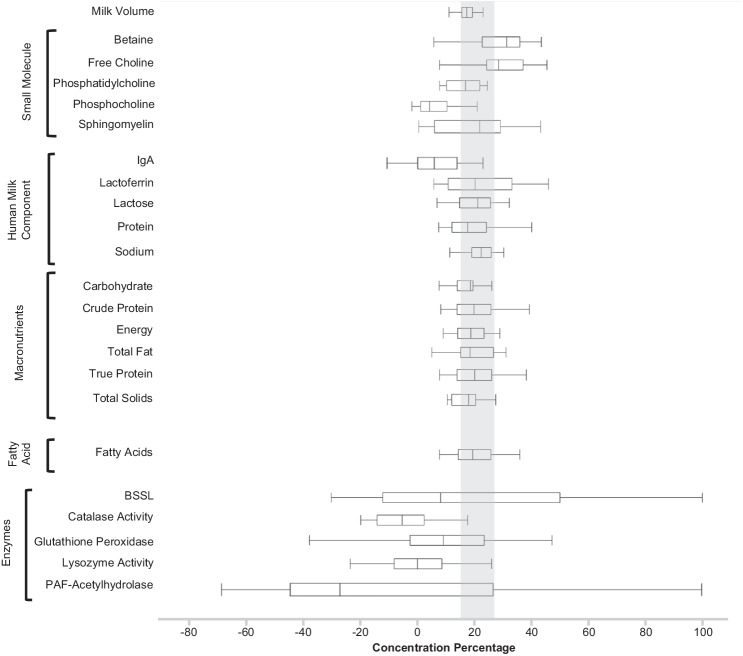
Fresh Human Milk Component Percent Concentration Change compared to Human Milk Percent Concentration Change. Box Plot (center value median) of nutrient concentration, *N*_con_, compared to the average mother’s milk concentration, *HM*_con_ (gray bar denotes expected nutrient concentration, *N*_con_).

The average percentage reduction in fresh HM volume (${{HM}}_{{con}}$) after passive osmotic concentration using the HMC device was 16.3% ± 3.8% (Fig. 2). Assuming no nutrient loss during passive osmotic concentration, the percentage increase in the content of an HM component (${N}_{{con}}$) is expected to be greater than ${{HM}}_{{con}}$ scaled by the ratio of ${V}_{{initial}}$ to$\,{V}_{{final}}$, as illustrated by the following comparison (where *N*_abs_ is the mass of the nutrient and ${V}_{{initial}}$ is 75 mL):

$$\frac{\left|\frac{{N}_{{abs}}}{{V}_{{final}}}-\frac{{N}_{{abs}}}{{V}_{{initial}}}\right|}{\frac{{N}_{{abs}}}{{V}_{{initial}}}}x100={N}_{{con}}$$

$$\frac{\left|\left(\frac{1}{{V}_{{final}}}-\frac{1}{{V}_{{initial}}}\right)\right|}{\frac{1}{{V}_{{initial}}}}x100$$

$$\frac{\left|{V}_{{initial}}-{V}_{{final}}\right|}{{V}_{{final}}}x100=\frac{{\Delta }_{{vol}}}{{V}_{{final}}}x100={N}_{{con}}$$

$$\frac{{V}_{{initial}}}{{V}_{{initial}}}\frac{{\varDelta }_{{vol}}}{{V}_{{final}}}x100=\frac{{V}_{{initial}}}{{V}_{{final}}}\left(\frac{{\Delta }_{{vol}}}{{V}_{{initial}}}x100\right)=\frac{{V}_{{initial}}}{{V}_{{final}}}\left({{HM}}_{{con}}\right)={N}_{{con}}$$

$$\frac{{V}_{{initial}}}{{V}_{{final}}}\left({{HM}}_{{con}}\right)={N}_{{con}}$$

$$\frac{{V}_{{initial}}}{{V}_{{final}}} > 1$$

Therefore,

$${N}_{{con}} > {{HM}}_{{con}}$$

**Fig. 3:**
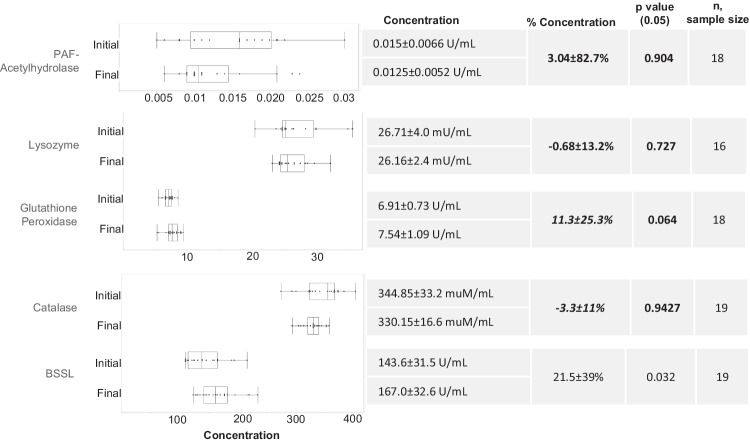
Comparison of initial and final concentration of component—box plot (center value median) of enzyme activity concentration, average initial/final concentration, % concentration, paired *t* test ([Component_final_]>[Component_initial_], and sample size. ***Bold*** denotes nutrient concentration less than anticipated HM volume concentration. Bold *p* value denotes concentration of nutrient was not significant.

**Fig. 4:**
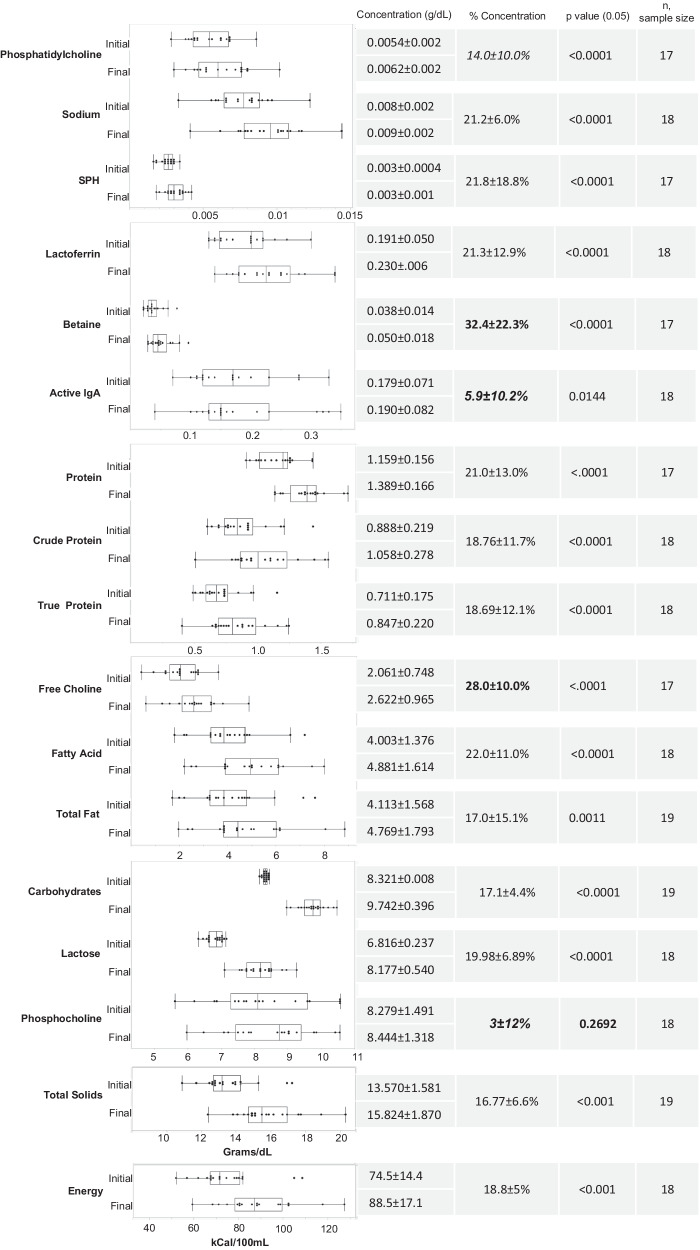
Comparison of initial and final concentration of nutrients—box plot (center value median) of nutrients concentration, average initial/final concentration, % concentration, paired *t* test ([Nutrient_final_]>[Nutrient_initial_], and sample size. Bold denotes nutrient concentration less than anticipated HM volume concentration. Bold denotes nutrient concentration greater than anticipated HM volume concentration. Bold *p* value denotes concentration of nutrient was not significant.

**Fig. 5:**
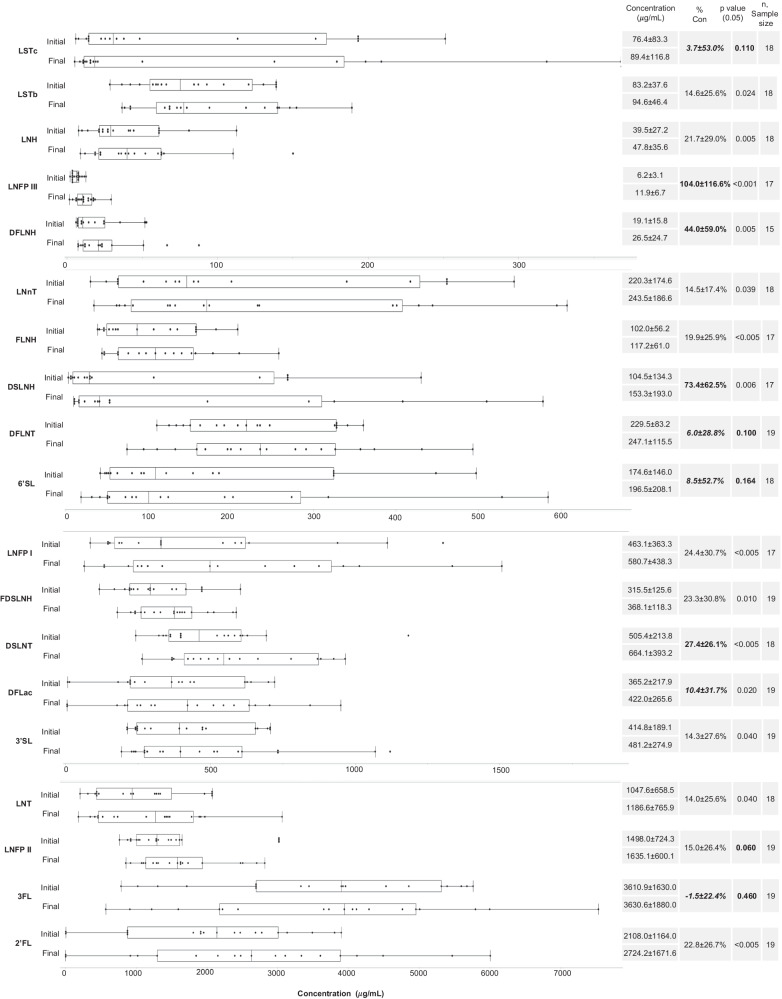
Comparison of initial and final concentration of HMOs—box plot (center value median) of HMO concentration (μg/mL), average initial/final concentration, % concentration, paired *t* test ([HMO_final_]>[HMO_initial_], and sample size. Bold denotes nutrient concentration less than anticipated HM volume concentration. Bold denotes nutrient concentration greater than anticipated HM volume concentration. Bold *p* value denotes concentration of nutrient was not significant.

Given the average ${{HM}}_{{con}}$ of $16.3 \% \pm 3.8 \% ,\,{N}_{{con}}$ values in the range of 14–25% were expected. Figure 1 compares the ${N}_{{con}}$ values of each analyzed HM component with ${{HM}}_{{con}}$. As shown in Figs. 3–5, ten of the 41 analyzed HM components did not differ significantly (*p* > 0.05) between unconcentrated and concentrated HM: PAF acetylhydrolase activity, lysozyme activity, glutathione peroxidase activity, catalase activity, phosphocholine concentration, and the concentrations of the HMOs: LSTc, DFLNT, 6’SL, LNFP II, and 3FL.

### Enzyme activity and nutrients

The box plots in Figs. 3 and 4 present the mean and standard deviation (SD) of enzyme activities and nutrient concentrations in HM before and after passive osmotic concentration. Among the five enzymes assayed, only the increase in BSSL activity was significant (21.5% ± 39%; *p* < 0.05) (Fig. 3). The increase in BSSL activity was also within the expected range.

The concentrations of all nutrients analyzed by MIRIS, i.e., carbohydrates, crude protein, energy, total fat, true protein, and total solids, increased significantly (*p* < 0.05) after treatment of HM with the HMC device (Figs. 3 and 4). All increases were within the expected range. The concentrations of sodium, protein, lactose, lactoferrin, and active IgA also increased significantly (*p* < 0.05) after passive osmotic concentration (Fig. 4). All increases were within the expected range with the exception of the increase in the concentration of active IgA (5.9 ± 10.2%), which was smaller than expected.

The concentrations of total fatty acids and all small molecules except phosphocholine increased significantly (*p* < 0.05) after passive osmotic concentration (Fig. 4). The increases in total fatty acids and SPH were within the expected range. The increases in betaine and free choline were greater than expected (32.4% ± 22.3% and 28.3% ± 9.6%, respectively). The failure to observe significant enrichment of phosphocholine may be attributable to the poor reproducibility of the HPLC-MS/MS assay for phosphocholine.

### Human milk oligosaccharides

As shown in Fig. 5, the concentrations of 14 of the 19 analyzed HMOs increased significantly (*p* < 0.05) after passive osmotic concentration. The exceptions were LSTc, DFLNT, 6’SL, LNFP II and 3FL. 3FL was the single HMO to decrease in concentration though this was not significant. Among the other 14 HMOs, the increases in the concentrations of LSTb, LNH, LNnT, FLNH, LNFP I, FDSLNH, 3’SL, LNT, and 2’FL were within the expected range. The concentrations of LNFP III, DFLNH, DSLNH, and DSLNT increased more than expected, whereas the concentration of DFLac increased less than expected.

### pH and osmolality

The mean pH of concentrated HM (7.08 ± 0.27) was lower than that of unconcentrated HM (7.37 ± 0.28), but this difference was not significant (*p* > 0.05). The mean osmolality increased significantly (*p* < 0.05) by 33%, from 295 ± 3.44 mOsm in unconcentrated HM to 392 ± 28.7 mOsm in concentrated HM.

### Total cell viability

The mean percentage of live cells did not differ significantly between unconcentrated HM and concentrated HM (7.63% versus 5.68%, *p* > 0.05).

## Discussion

The findings support the ability of the passive osmotic HMC device to concentrate fresh HM components in a novel point-of-care process. The osmolality and pH of the concentrated HM were within typical neonatal feeding parameters, supporting feeding tolerance. Among the 41 analyzed HM components, the concentrations of 31 were significantly increased by passive osmotic concentration; none were significantly decreased. The samples increased on average by 20% in kcal and protein with an average baseline of 22 kcal/oz with 1.16 gm protein/dl to post HM concentration 26 kcal/oz with 1.39 gm protein/dl.

Point-of-care concentration of MOM may be an alternative to adding fortifiers to MOM. Due to the high digestibility of MOM, concentrated MOM feedings may have greater absorption than formula/fortified feedings. Increasing the transfer of bioactive molecules, antioxidant capacity, and total nutrients via MOM may improve the short- and long-term health of preterm infants, which could have substantial economic and public health benefits.

Moreover, the point-of-care passive osmotic concentration of MOM using the HMC device avoids heat or pressure damage to fragile components that are unique to MOM, such as living cells, digestive enzymes, and bioactive molecules. Commercial fortifiers have been shown to reduce the activity of some enzymes and immune components in human milk [23, 34]. By avoiding or reducing the use of fortifiers, feeding concentrated MOM may ensure that these enzymes are most active in the neonatal gut versus interacting with a fortifier while in a storage container. A recent study indicated that most mothers of preterm infants in NICUs who are physically able to and desire to establish lactation can establish an adequate maternal milk supply [35–37]. In a survey of parents whose babies had been in NICUs, the majority associated their baby’s feeding fortification in the NICU with feelings of powerlessness, inadequacy, disappointment, and worry [38]. Conversely, a previous study documented that NICU mothers felt empowered by providing their own milk for feeding their preterm babies in the NICU [39].

HM concentration is a paradigm change that warrants further study due to its potential to provide greater benefits than current feeding models. Importantly, HM concentration via passive osmosis may increase the efficiency of the NICU feeding preparation workflow rather than diminish it. The passive osmotic membrane technology comprises components that are already used in neonatal feeding and care and are similar to the way plants naturally draw water into the xylem via osmosis. Osmosis has long been used to filter water and to concentrate bovine dairy products under pressure. Water removal from HM via passive osmosis may be far less risky than feeding preterm infants bovine-derived formulas.

### Limitations and future directions

The findings of this study are subject to limitations of the study design and methodologies. Because the HMC device is intended for point-of-care use, fresh HM samples were analyzed in this study, which limited the sample size. Moreover, because the fresh HM samples were provided by volunteer donors, the expression conditions and storage times varied, which may explain the low cell viability in the unconcentrated samples. In some cases, the storage time exceeded 20 h, whereas the average viability of cells in HM is 8 h. Nonetheless, cell viability did not differ significantly between unconcentrated and concentrated HM, indicating that passive osmotic concentration by the HMC device did not reduce cell viability. Follow-up studies collecting larger numbers of fresh HM samples and information on the lactation cycles of the HM donors are required.

Among methodological limitations, the accuracy of the Miris HMA when used with concentrated HM samples has not been validated. Two different methods for measuring protein (BCA and infrared) indicated similar degrees of concentration by the device, suggesting that Miris can accurately measure higher protein concentrations in HM. Structural commonalities in HMOs may explain why some HMOs did not increase as much as other HMOs in concentrated HM samples, which may be clarified by further study. For example, 6’SL and LSTc each carry a 2–6 sialic acid linked to a terminal galactose, which introduces a specific negative charge that may interact with the membrane and be retained. In addition, the measurements of total fatty acid concentration were highly variable, which may have been due to the presence of concentrations outside the linear range of the GC-FID assay. The GC-FID assay was shown to be linear between 3.75 and 6.1 g/dL, whereas the total fatty acid concentration in the unconcentrated HM samples ranged from 1.7 g/dL to 7.6 g/dL. However, the linear dynamic range of flame ionization detection in GC is very wide, i.e., up to 7 orders of magnitude; thus, the linearity of the GC assay can likely be extrapolated to cover the entire range of total fatty acid content in HM samples. Finally, in some HM samples, the concentrations of HM components did significantly change after incubation with the HMC device. This phenomenon was particularly common for phosphocholine. Phosphocholine is more challenging to analyze by HPLC-MS/MS than free choline or betaine because it is simultaneously positively and negatively charged, and the negatively charged phosphate group is known to interact with exposed cations in the HPLC’s stainless steel tubing, especially at low concentrations. The CV was ~10% for phosphocholine, in contrast to ~3% for free choline or betaine. Further studies of the interactions of specific HM components of different charges and sizes with osmotic membranes are needed.

Future studies should also examine other beneficial HM components that may be enriched by point-of-care passive osmotic concentration of HM. Hormone content in MOM and DHM is an area of expanding research [18]. One example, melatonin is provided exclusively by MOM or DHM until endogenous production of melatonin begins in infants at ~90 days of life. Metabolites of melatonin have scavenger functions and can ‘induce an antioxidant cascade that quenches ten radical products…making it more efficient than glutathione’ [40]. There are conflicting research findings on the impact of pasteurization techniques on melatonin levels in donor HM. In addition, the impact of point-of-care passive osmotic concentration of fresh MOM (<4 h old) on neonatal growth rates warrants further investigation. The passive osmotic process may amplify the benefits of feeding preterm infants fresh MOM. Additionally, pH is logarithmic, so the shift noted should be further investigated to evaluate if increasing enzymatic activity may reduce pH. Filatava et al found variations in pH of MOM based on lactation stage and maternal diet; and also that fortified MOM had a lower pH than MOM at baseline [41]. Fortified MOM’s pH decreased over storage time in Filatava’s study, and it was noted that common HMF products are noted to be acidic (pH 5.79–5.94), whereas the concentrated MOM in our study had pH levels closer to unfortified MOM. This indicates that concentrated MOM may have less change in pH, which may improve feeding preterm infant tolerance despite increased nutrient density by volume, and further study is indicated to assess the clinical impact of non-significant changes in pH.

Future studies will focus standard FDA-indicated safety analysis to confirm lack of leachables, toxicology, and bacteria analysis as well as evidence base for the improved clinical outcomes and further integration into donor milk banks.

## Conclusion

The COVID-19 pandemic, the 2022 US infant formula supply chain crisis, and recent formula recalls in 2024 illuminated the importance of MOM as an ideal preterm infant food that provides immunologically protective benefits and nutrition [42]. Point-of-care passive osmotic concentration of MOM using the HMC device may be a valuable novel approach to support and enhance the impact of MOM in neonatal feeding. Exclusive breastfeeding/MOM feeding and extended lactation through the first year of life have proven benefits for both preterm infants and their mothers [43].

Point-of-care passive osmotic concentration of MOM may empower the mothers of preterm infants by enabling them to feed their babies more mother’s own milk and by reinforcing the positive impact of lactation.
